# Greater Adherence to Cardioprotective Diet Can Reduce Inflammatory Bowel Disease Risk: A Longitudinal Cohort Study

**DOI:** 10.3390/nu14194058

**Published:** 2022-09-29

**Authors:** Tian Fu, Shuyu Ye, Yuhao Sun, Lintao Dan, Xiaoyan Wang, Jie Chen

**Affiliations:** 1 Department of Gastroenterology, The Third Xiangya Hospital, Central South University, 138 Tongzipo Road, Changsha 410013, China; 2 Center for Global Health, Zhejiang University School of Medicine, 866 Yuhangtang Road, Hangzhou 310058, China

**Keywords:** nutrition, cardioprotective diet, inflammatory bowel disease, diet pattern

## Abstract

Background: The cardioprotective diet was reported to be associated with several chronic cardiometabolic diseases through an anti-inflammation effect. However, the association between the cardioprotective diet and the risk of inflammatory bowel disease (IBD) was unclear and deserved to be further explored. Methods: We calculated the cardioprotective diet score based on the consumptions of seven common food groups using the validated food frequency questionnaire data in the UK Biobank. Incident IBD was ascertained from primary care data, inpatient data, and the death registry. Cox proportional hazard models were used to estimate the hazard ratios (HRs) and 95% confidence intervals (CIs) for associations between the cardioprotective diet score and the risk of IBD. Results: During a mean follow-up of 12.1 years, we documented 2717 incident IBD cases, including 851 cases of Crohn’s disease and 1866 cases of ulcerative colitis. Compared to participants with a cardioprotective diet score of 0–1, we observed a decreased risk of IBD among participants with cardioprotective diet scores of 3 (HR 0.85, 95% CI 0.73–0.99), 4 (HR 0.84, 95% CI 0.72–0.98), and 5–7 (HR 0.77, 95% CI 0.66–0.89) (*p*-trend < 0.001). Conclusions: A greater adherence to the cardioprotective diet was associated with a lower risk of IBD. Our finding highlighted the importance of focusing on the cardioprotective diet to prevent IBD.

## 1. Introduction

Inflammatory bowel disease (IBD) is a recurrent and remitting gastrointestinal chronic inflammatory disorder that comprises Crohn’s disease (CD) and ulcerative colitis (UC) [1]. The prevalence of IBD has been increasing and is expected to be as high as 1% in most Western countries by 2030 [2]. Genetic susceptibility and multiple environmental factors seem to contribute together to the development of IBD; however, the specific pathogenesis was unclear [3]. As one of the modifiable risk factors, diet was thought to play an essential role in IBD by altering inflammation, the gut microbiome, epithelial barrier function, and intestinal immunity [3]. Compared to single food groups or nutrients, studies on overall dietary quality preserved the complexities and synergism [4] between diets and have been increasingly used to study IBD [5].

The cardioprotective diet is a measurement of diet quality proposed recently for cardiometabolic health that consists of food items including fruits, vegetables, whole grains, fish, refined grains meat, wine, tea, and curry, etc. [6]. Chronic diseases such as coronary heart disease [6], diabetes [7], and dementia [8] were reported to benefit from the cardioprotective diet. In parallel, there is mounting evidence for the critical role of these chronic diseases and corresponding characteristic metabolites in IBD [9,10,11]. In addition, inflammation was reported to play important role in the relationship between the cardioprotective diet and cardiometabolic diseases, as well as between diet and IBD [3,6]. A series of reviews and epidemiological studies have identified the effects of food groups comprising the cardioprotective diet such as fruits and vegetables, fish, processed meat, and grains on IBD via anti-inflammatory or pro-inflammatory pathways [12,13,14,15].

Nevertheless, there has been no examination of the association between cardioprotective diet scoring and the risk of incident IBD. Here, we conducted a longitudinal cohort study in the UK Biobank using detailed and validated dietary data to study the association between the cardioprotective diet and the risk of IBD.

## 2. Materials and Methods

### 2.1. Study Population

The UK Biobank is a cohort study that includes over 500,000 participants aged 40–69 years who enrolled in 2006–2010. At recruitment, participants attended one of the twenty-two assessment centers across England, Wales, and Scotland, and were asked to complete a touchscreen questionnaire, a verbal interview, physical measurements, and a biological sample collection. Ethical approval for the UK Biobank was granted by the North West-Haydock Research Ethics Committee (REC reference: 21/NW/0157). More available details and resources are depicted on the website of UK Biobank (https://www.ukbiobank.ac.uk (accessed on 10 September 2022)). Participants included in the current study provided written informed consent.

**Figure 1:**
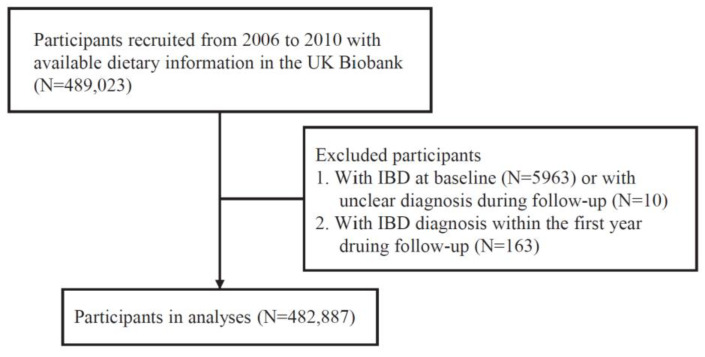
Flowchart of study participants in the UK Biobank. IBD, inflammatory bowel disease.

Participants with available dietary information were included (N = 489,023). In addition, participants with IBD at baseline (N = 5963) or an uncertain IBD diagnosis during follow-up (N = 10) were excluded. We also excluded participants with a diagnosis of IBD within the first year during follow-up (N = 163) to reduce reverse causation. Finally, 482,887 participants were included in the analysis (Figure 1).

### 2.2. Dietary Assessment

Dietary information was obtained from the food frequency questionnaire (FFQ) at baseline, which was validated with moderate to substantial reproducibility in main dietary variables [16]. The questionnaire asked about the consumption of commonly consumed food groups in the past year; the corresponding answer included six possible responses: “never”, “less than once a week”, “once a week”, “2–4 times a week”, “5–6 times a week”, or “once or more daily” for frequency or the specified serving size (slices/bowls/teaspoons per day/week) for average consumption. 

The cardioprotective diet, which was based on recommendations by the American Heart Association [6], defines the ideal food intake [17] for cardiometabolic health. According to previous studies, the cardioprotective diet score used in the current study was developed based on available food items collected by the FFQ [7,8,18] and included seven food groups: fruits (fresh or dried), vegetables (cooked or raw), whole grains, fish (oily or non-oily), refined grains, processed meats, and unprocessed meats (poultry, mutton, beef, pork). Specifically, each food group was divided into two categories and one point was given when meeting the intake goal: fruits ≥ 3 servings/day, vegetables ≥ 3 servings/day, whole grains ≥ 3 servings/day, fish ≥ 2 servings/week, refined grains ≤ 2 servings/day, unprocessed meat ≤ 2 servings/week, and processed meat ≤ 1 serving/week. The calculated cardioprotective diet score ranged from 0–7, with higher scores representing a greater adherence to the cardioprotective diet. We further divided the score into five categories (scores of 0–1, 2, 3, 4, and 5–7) to ensure each group had enough participants.

### 2.3. Assessment of Outcome 

Incident IBD was identified through linkage to the inpatient data (International Classification of Diseases Ninth and Tenth editions (ICD-9 and ICD-10, respectively)), coded primary care data (read codes were converted into ICD codes), and death registry (ICD-10). Specifically, IBD was defined as CD (ICD-9: 555; ICD-10: K50) and UC (ICD-9: 556; ICD-10: K51). The health outcome data were available up to 28 February 2018 for Wales, 31 July for Scotland and 30 September for England, respectively. 

### 2.4. Assessment of Covariates

The potential confounders were selected based on prior knowledge and previous studies [12,19,20] and included age at recruitment, sex (female or male), ethnicity (white and others), education level (college and above or high school and below), Townsend deprivation index (TDI; tertile into low, moderate, and high deprivation), physical activity levels assessed via the International Physical Activity Questionnaire (IPAQ) [21] (low, moderate, or high), smoking status (never, previous, or current), alcohol drinking status (non-current or current), and body mass index (BMI in kg/m^2^). Considering possible confounding of medication, comorbidities, and inflammatory indicators in IBD risk, [19] baseline medication use including non-steroidal anti-inflammatory drugs, proton pump inhibitors and antibiotics, serum C-reactive protein, and comorbidities represented by the Charlson Comorbidity Index [22] were also included as covariates. Other food groups proposed as components in the cardioprotective diet but not described in detail in the FFQ [8] in the UK Biobank (vegetable oils, sugar-sweetened beverages, and dairy products) were also selected as covariates. Ideal intake was defined as ≥2 servings/week for dairy products and vegetable oils and 0 servings/day for sugar-sweetened beverages. Participants meeting the ideal intake received 1 point and 0 points otherwise. TDI was a composite measure of deprivation based on the preceding national census output areas and composed of unemployment, non-car ownership, non-home ownership, and household crowding. A lower TDI score indicated a higher level of socioeconomic status. Medication use was collected via the touchscreen questionnaire and the verbal interview at baseline; the Charlson Comorbidity Index was constructed using multiple comorbidities according to ICD codes and measured comorbid disease status. The specific classifications can be found in Supplementary Table S1. For continuous and categorical covariates, we filled missing values with the mean or into the most populated category, respectively. If covariates had a missing rate of >5% [7], the missing data were coded as a separate category.

### 2.5. Statistical Analyses

Baseline characteristics were summarized according to the five categories of cardioprotective diet scores (scores of 0–1, 2, 3, 4, and 5–7). The continuous and categorical variables were presented in the form of means (standard deviations (SDs)) and numbers (percentages), respectively. We used Cox proportional hazard models to estimate the hazard ratios (HRs) and 95% confidence intervals (CIs) for associations between the cardioprotective diet scores and the risk of IBD. Person-years were calculated from the baseline date to the date of diagnosis, death, loss, or the end of follow-up, whichever came first. Two multivariable-adjusted models were used to evaluate the associations. The minimally adjusted model was adjusted for age, the square of age, ethnicity, and sex; the fully adjusted model was further adjusted for education levels, physical activity levels, alcohol drinking status, smoking status, TDI, and BMI. We also tested and verified the proportional hazard assumption using Schoenfeld residual methods [23]. Testing for trends across different cardioprotective diet scores was conducted by treating assigning values as a continuous variable. In addition, we separately examined the associations between the cardioprotective diet scores and the risks of CD and UC. 

For analyses of individual components of the cardioprotective diet, we explored the associations with IBD risk in models with a single food group and seven food groups that were mutually adjusted. To evaluate the potential effects of age (≤60 or >60 years), sex (female or male), ethnicity (white and other), education levels (college and above or high school and below), smoking status (never, previous, or current), alcohol drinking status (non-current or current), TDI (low, moderate, or high), and physical activity levels (low, moderate, or high), we tested the P-interaction between the cardioprotective diet scores and each covariate and conducted subgroup analyses. 

Several sensitivity analyses based on the fully adjusted model were conducted to verify the robustness of primary results: (1) further adjusted for other food groups including vegetable oils, sugar-sweetened beverages, and dairy products; (2) further adjusted for baseline medication use including non-steroidal anti-inflammatory drugs, proton pump inhibitors, and antibiotics; (3) further adjusted for serum C-reactive protein; (4) further adjusted for the Charlson Comorbidity Index; (5) participants with colorectal cancer at baseline excluded; and (6) missing values of covariates reprocessed with multiple imputations to minimize the effects of the imputing method.

All statistical analyses were performed with R 4.1.1. A two-sided *p*-value of <0.05 was considered statistically significant.

## 3. Results

### 3.1. Baseline Characteristics

Baseline characteristics of the study population are shown in Table 1. A total of 482,887 participants were included with a mean (SD) age of 56.56 (8.09) years and mean (SD) BMI of 27.43 (4.78), of which 54.4% were female and 94.9% were of white ethnicity. Compared to participants with cardioprotective diet scores of 0–1, those with higher scores were more likely to be female and non-smokers with a lower BMI, more physical activities, and higher educational levels. During a mean (SD) follow-up of 12.1 (1.9) years, we documented 2717 incident IBD cases (46.4 cases/100,000 person-years) that included 851 cases of CD (14.5 cases/100,000 person-years) and 1866 cases of UC (31.9 cases/100,000 person-years).

### 3.2. Primary Analyses

**Figure 2:**
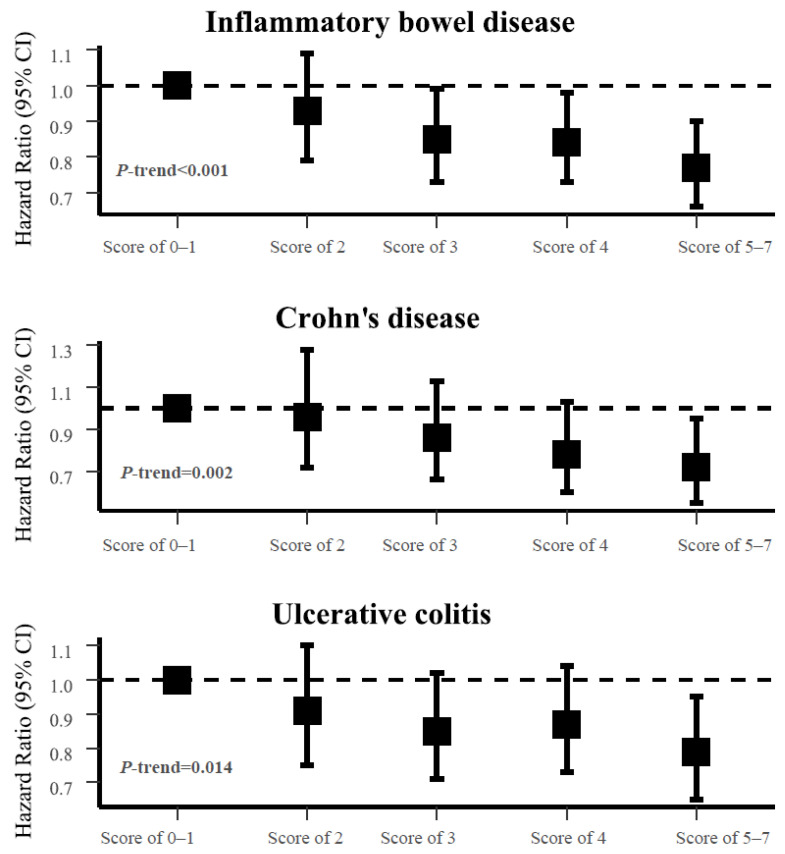
Associations between cardioprotective diet scores and risk of inflammatory bowel disease, Crohn’s disease, and ulcerative colitis. HRs and 95% CIs were calculated using the Cox model adjusted for sex, age, age squared, ethnicity, education, TDI, physical activity, smoking status, alcohol drinking status, and BMI. Abbreviations: CI, confidence interval; HR, hazard ratio; BMI, body mass index; TDI, Townsend deprivation index.

We found an inverse association between the cardioprotective diet scores and the IBD risk (Table 2, Figure 2). Specifically, in the minimally adjusted model adjusted for age, age squared, sex, and ethnicity, the participants with cardioprotective diet scores of 3 (HR 0.78, 95% CI 0.67–0.91, *p* = 0.002), 4 (HR 0.75, 95% CI 0.64–0.87, *p* < 0.001), and 5–7 (HR 0.65, 95% CI 0.56–0.76, *p* < 0.001) (*p*-trend < 0.001) were associated with a decreased IBD risk compared to those with a score of 0–1. The HR was 0.85 (95% CI 0.73–0.99, *p* = 0.041) for participants with a cardioprotective diet score of 3, 0.84 (95% CI 0.72–0.98, *p* = 0.027) for a score of 4, and 0.77 (95% CI 0.66–0.89, *p* = 0.001) for score of 5–7 (*p*-trend < 0.001) compared to a score of 0–1 in the fully adjusted model further adjusted for TDI, education, smoking, alcohol drinking, physical activity, and BMI. Consistent inverse associations (Figure 2, Supplementary Table S2) between the cardioprotective diet scores and the risk of CD (HR-compared score of 5–7 with a score of 0–1 = 0.72, 95% CI 0.55–0.95, *p*-trend = 0.002) and UC (HR-compared score of 5–7 with a score of 0–1 = 0.79, 95% CI 0.65–0.95, *p*-trend = 0.014) were also observed. 

### 3.3. Secondary and Sensitivity Analyses

The effects of single food groups comprising the cardioprotective diet are presented in Supplementary Table S3. Compared to a poor intake, ideal intakes of fruits (HR 0.88, 95% CI 0.81–0.95, *p* = 0.001), vegetables (HR 0.86, 95% CI 0.78–0.94, *p* = 0.001), and whole grains (HR 0.87, 95% CI 0.77–0.98, *p* = 0.019) were associated with a decreased risk of IBD. When mutually adjusted for seven food groups in the model, the associations were also observed for fruits (HR 0.90, 95% CI 0.83–0.98, *p* = 0.010) and vegetables (HR 0.88, 95% CI 0.80–0.97, *p* = 0.013). We did not observe associations of the consumption of fish (HR 0.99, 95% CI 0.91–1.07, *p* = 0.744), refined grains (HR 0.96, 95% CI 0.87–1.06, *p* = 0.376), processed meat (HR 1.00, 95% CI 0.92–1.09, *p* = 0.943), or unprocessed meat (HR 1.04, 95% CI 0.94–1.16, *p* = 0.413) with IBD risk. In the subgroup analyses, the main results did not differ substantially and no multiple interactions were observed (all P for interaction > 0.05) (Supplementary Tables S4–S6). Participants who were male (HR-compared score of 5–7 with a score of 0–1 = 0.77, 95% CI 0.63–0.94, *p* for trend = 0.004) and more than 60 years old (HR-compared score of 5–7 with a score of 0–1 = 0.73, 95% CI 0.57–0.93, *p* for trend = 0.001) with white ethnicity (HR-compared score of 5–7 with a score of 0–1 = 0.74, 95% CI 0.63–0.87, *p* for trend < 0.001), a high TDI (HR-compared score of 5–7 with a score of 0–1 = 0.74, 95% CI 0.59–0.94, *p* for trend = 0.004), lower educational levels (high school and below) (HR-compared score of 5–7 with a score of 0–1 = 0.75, 95% CI 0.63–0.90, *p* for trend = 0.001), previously smoked (HR comparing score of 5–7 with score of 0–1 = 0.66, 95% CI 0.52–0.85, *p* for trend < 0.001), and current alcohol consumers (HR-compared score of 5–7 with a score of 0–1 = 0.75, 95% CI 0.64–0.88, *p* for trend < 0.001) were more likely to have a decreased risk of IBD (Supplementary Tables S4–S6).

The main findings remained stable in the sensitivity analyses (Supplementary Tables S7 and S8). Compared to participants with score of 0–1, those with the highest cardioprotective diet scores (5–7) were associated with a decreased risk of IBD when further adjusted for other food groups (HR-compared score of 5–7 with a score of 0–1 = 0.76, 95% CI 0.65–0.89, *p*-trend < 0.001), further adjusted for medication use (HR-compared score of 5–7 with a score of 0–1 = 0.78, 95% CI 0.67–0.91, *p*-trend < 0.001), further adjusted for serum C-reactive protein (HR-compared score of 5–7 with a score of 0–1 = 0.78, 95% CI 0.67–0.91, *p*-trend = 0.001), further adjusted for the Charlson Comorbidity Index (HR-compared score of 5–7 with a score of 0–1 = 0.77, 95% CI 0.66–0.90, *p*-trend < 0.001), participants with colorectal cancer at baseline were excluded (HR-compared score of 5–7 with a score of 0–1 = 0.77, 95% CI 0.66–0.90, *p*-trend < 0.001), or the imputing method was replaced (HR-compared score of 5–7 with a score of 0–1 = 0.80, 95% CI 0.67–0.96, *p*-trend = 0.003).

## 4. Discussion

In this large longitudinal cohort study among participants in the UK Biobank, we observed that higher cardioprotective diet scores were associated with a lower risk of IBD. Similar associations were also found in the risks of CD and UC. Additionally, intake of specific components of the cardioprotective diet, including fruits and vegetables, was inversely associated with IBD risk.

Although the cardioprotective diet has not been studied in IBD, previous epidemiological studies have linked the cardioprotective diet and similar diets to a risk of chronic disease [24]. Chronic diseases such as coronary heart disease [11] and diabetes [25] were reported to relate to IBD in a series of studies based on genetic data and experimental data; in parallel, several overlapping risk factors and biomarkers were reported in the development of cardiometabolic disease and IBD [26,27]. Therefore, the current analysis filled the research gap on the cardioprotective diet and IBD. Associations between dietary patterns with similar food groups to the cardioprotective diet and IBD risk have been extensively explored, with our study showing consistent findings. Evidence from three large prospective cohorts in the United States that included 166,903 women and 41,931 men [28] demonstrated that individuals with a high-inflammatory-potential diet including processed meat, red meat, fish, vegetables, and refined grains might have an increased risk of CD within over 4,949,938 person-years of follow-up. A prospective population-based cohort based on 125,445 participants (224 UC and 97 CD occurred) over a 14-year follow-up revealed that a dietary pattern [29] characterized by low vegetable and fruit consumption and a low diet-quality score comprising vegetables, fruits, whole grain products, and red and processed meat was associated with a higher risk of CD development, while a pattern consisting of red meat, poultry, and processed meat was associated with an increased likelihood of UC development. Another study conducted in the NutriNet-Santé Cohort observed a weak association between dietary patterns [30] that was correlated with vegetables, fruits, whole grain products, processed meat, fish, and IBD risk. In addition, prior studies showed an association between individual food groups and nutrients included in the cardioprotective diet and the risk of IBD [12,14,15]. Notably, our results suggested a protective effect of fruits, vegetables, and whole grains in the risk of IBD, which was in line with these studies. However, no significant associations were observed between meats, refined grains, and fish in our study. One of the reasons for this difference may have been the complexities and synergism between diets. On the other hand, diet patterns consisting of variable food groups and specific serving sizes could have different effects on the development of IBD. Overall, the current study, which concentrated on the cardioprotective diet, found an inverse association with IBD risk, which provided references for preventing IBD.

Several mechanisms may explain the current findings. First, the anti-inflammatory potential of the cardioprotective diet may partly explain the inverse association. Inflammation and oxidative stress caused by diet played critical roles in IBD [17]; cardioprotective diet supplementation was found to reduce oxidative stress, inflammation, and mucosal damage in animal colitis models [31]. Several dietary intervention studies also demonstrated that a diet that improved cardiometabolic health could lead to changes in inflammatory markers, [32] immune cell populations, and oxidative stress [33]. The current findings were kept stable when further adjusted for serum C-reactive protein, indicating other non-inflammatory pathways that deserved to be explored. Secondly, metabolic indicators such as lipid metabolites [34] and BMI [35] improved by the cardioprotective diet were associated with the risk of IBD, suggesting that the protective roles of IBD may be related to decreased metabolic comorbidities. We observed a lower BMI in participants with higher cardioprotective diet scores but no effects of multiple baseline comorbidities on the results were seen. Finally, previous in vivo and in vitro studies revealed potential pathways through which dietary components of cardioprotective diet can have an effect on IBD. For example, dietary fiber from fruits and vegetables can be fermented by anaerobic bacteria to produce short-chain fatty acids (SCFAs), which play an important role in the pathogenesis of IBD via anti-inflammation and improvement of the gut barrier function [36]. The dietary grain source is associated with circulating inflammation markers and the abundance of gut microbiota that produce SCFAs, with whole grains being anti-inflammatory but refined grains being pro-inflammatory [37]. This is consistent with the results of randomized dietary intervention studies [38] and our study showing that whole grains were associated with a lower IBD risk. In addition, fish that are high in omega-3 polyunsaturated fatty acids can influence the intestinal microbiota and immune system [39]. Excessive consumption of red meat and processed meat can increase the concentration of trimethylamine N-oxide [40], which has been proven to be associated with inflammation. Overall, the interaction between the cardioprotective diet and diseases and IBD warrants more epidemiologic and experimental evidence. 

There were several strengths to our study. First, to our knowledge, this was the first study to explore the relationship between the cardioprotective diet and risk of IBD. We collected detailed information from the cohort’s large sample size and found enough incident cases to examine the associations, facilitate the subgroup analyses, and minimize the effects of confounding factors. The incidence of IBD was 46.4 per 100,000 person years in the current study; according to previous studies, the incidence of IBD in the UK ranged from 28.6 to 69.5 per 100,000 person years [41,42,43]. The disparity from other studies may be explained by the older demographic in our study. In addition, dietary information collected via the FFQ in the UK Biobank collected the dietary intake from the past year, reflecting the long-term dietary habits and reduced bias brought about by changeable dietary habits, which were reported to have moderate to substantial reproducibility [16]. Second, the detailed outcome data were collected from multiple sources including primary care data, inpatient data, and the death registry. Third, we considered the diagnosis delay for IBD and excluded participants diagnosed with IBD within the first year during follow-up to reduce potential reverse causation. A multi-center cohort study among the European population demonstrated that the median delay in the diagnosis of IBD was 3 months (interquartile range (IQR) of 0.8–12) [44]. 

We acknowledge several limitations of our study as well. First, participants who followed a specific dietary pattern such as vegetarianism were not considered. Given the complex interactions between diets, more interventional studies are needed to further prove the current findings. Moreover, due to cohort limitations, our study was predominantly composed of a population of white ethnicity and middle age, so the effects of food groups and overall diet on the risk of IBD might be inconsistent in other populations; thus, caution is needed when interpreting the current findings. Finally, as in all observational research, residual confounding (e.g., total energy intake) and reverse causation could not be avoided.

In conclusion, the results of this large longitudinal cohort of the UK Biobank suggested associations between greater adherence to the cardioprotective diet and a lower risk of IBD. Although further epidemiological research among other populations and more experimental data are needed, our study highlighted the importance of focusing on the benefits of the cardioprotective diet to prevent IBD.

## Figures and Tables

**Table 1:** Characteristics of all participants based on different cardioprotective diet scores.

	Overall (n = 482,887)	Score of 0–1 (n = 32,526)	Score of 2 (n = 65,410)	Score of 3 (n = 110,015)	Score of 4 (n = 132,261)	Score of 5–7 (n = 142,675)
**Sex (%)**						
Female	262,889 (54.4)	8451 (26.0)	25,548 (39.1)	55,754 (50.7)	79,545 (60.1)	93,591 (65.6)
Male	219,998 (45.6)	24,075 (74.0)	39,862 (60.9)	54,261 (49.3)	52,716 (39.9)	49,084 (34.4)
**Age (mean (SD))**	56.56 (8.09)	54.48 (8.30)	55.43 (8.28)	56.05 (8.17)	56.82 (7.99)	57.70 (7.78)
**Ethnicity (%)**						
White	458,310 (94.9)	31,529 (96.9)	62,583 (95.7)	104,657 (95.1)	124,940 (94.5)	134,601 (94.3)
Others	24,577 (5.1)	997 (3.1)	2827 (4.3)	5358 (4.9)	7321 (5.5)	8074 (5.7)
**TDI (%)**						
High deprivation	160,948 (33.3)	13,091 (40.2)	23,890 (36.5)	36,878 (33.5)	42,128 (31.9)	44,961 (31.5)
Low deprivation	160,977 (33.3)	9173 (28.2)	20,182 (30.9)	36,636 (33.3)	45,425 (34.3)	49,561 (34.7)
Moderate deprivation	160,962 (33.3)	10,262 (31.6)	21,338 (32.6)	36,501 (33.2)	44,708 (33.8)	48,153 (33.8)
**Educational level (%)**						
College and above	156,463 (32.4)	6999 (21.5)	16,780 (25.7)	32,989 (30.0)	44,403 (33.6)	55,292 (38.8)
High school and below	326,424 (67.6)	25,527 (78.5)	48,630 (74.3)	77,026 (70.0)	87,858 (66.4)	87,383 (61.2)
**Smoking (%)**						
Current	50,141 (10.4)	6616 (20.3)	10,311 (15.8)	12,969 (11.8)	11,542 (8.7)	8703 (6.1)
Never	266,149 (55.1)	15,493 (47.6)	33,313 (50.9)	59,294 (53.9)	74,370 (56.2)	83,679 (58.7)
Previous	166,597 (34.5)	10,417 (32.0)	21,786 (33.3)	37,752 (34.3)	46,349 (35.0)	50,293 (35.3)
**Alcohol drinking (%)**						
Current	445,168 (92.2)	30,237 (93.0)	60,573 (92.6)	101,929 (92.7)	122,043 (92.3)	130,386 (91.4)
Non-current	37,719 (7.8)	2289 (7.0)	4837 (7.4)	8086 (7.3)	10,218 (7.7)	12,289 (8.6)
**Physical activity (%)**						
High	157,881 (32.7)	8830 (27.1)	17,954 (27.4)	32,329 (29.4)	43,165 (32.6)	55,603 (39.0)
Moderate	159,661 (33.1)	10,144 (31.2)	20,993 (32.1)	36,744 (33.4)	44,593 (33.7)	47,187 (33.1)
Low	73,615 (15.2)	6884 (21.2)	12,756 (19.5)	19,009 (17.3)	19,261 (14.6)	15,705 (11.0)
Miss	91,730 (19.0)	6668 (20.5)	13,707 (21.0)	21,933 (19.9)	25,242 (19.1)	24,180 (16.9)
**BMI (mean (SD))**	27.43 (4.78)	28.25 (4.91)	28.03 (4.88)	27.75 (4.82)	27.42 (4.75)	26.72 (4.60)

Abbreviations: SD, standard deviation; TDI, Townsend deprivation index; BMI, body mass index.

**Table 2:** Association between cardioprotective diet scores and risk of inflammatory bowel disease in multivariable models.

Cardioprotective Diet Scores	Cases	Person-Years	Minimally Adjusted Model ^1^		Fully Adjusted Model ^2^	
			HR (95% CI)	*p*-Value	HR (95%CI)	*p*-Value
**Score of 0–1**	235	390,687	Ref		Ref	
**Score of 2**	424	788,123	0.89 (0.76, 1.04)	0.146	0.93 (0.79, 1.09)	0.356
**Score of 3**	632	1,331,613	0.78 (0.67, 0.91)	**0.002**	0.85 (0.73, 0.99)	**0.041**
**Score of 4**	730	1,605,282	0.75 (0.64, 0.87)	**<0.001**	0.84 (0.72, 0.98)	**0.027**
**Score of 5–7**	696	1,738,044	0.65 (0.56, 0.76)	**<0.001**	0.77 (0.66, 0.89)	**0.001**
***p*-trend**				**<0.001**		**<0.001**

^1^ Minimally adjusted model: adjusted for age, age squared, sex, and ethnicity; ^2^ fully adjusted model: adjusted for age, age squared, sex, ethnicity, Townsend deprivation index, education, smoking, alcohol drinking, physical activity, and body mass index. Abbreviations: CI, confidence interval; HR, hazard ratio.

## Data Availability

Researchers can acquire the data and approval from the UK Biobank (www.ukbiobank.ac.uk (accessed on 10 September 2022)).

## Supplementary Materials

The following supporting information can be downloaded at: https://www.mdpi.com/article/10.3390/nu14194058/s1, Supplementary Table S1. Definition and classification of covariates; Supplementary Table S2. Association between cardioprotective diet and risk of Crohn’s disease and ulcerative colitis; Supplementary Table S3. Association between each component of cardioprotective diet and risk of IBD in models with single food group and all seven food groups mutually adjusted; Supplementary Table S4. Subgroup analyses of the associations between cardioprotective diet scores and risk of inflammatory bowel disease stratified by sex, age, and ethnicity; Supplementary Table S5. Subgroup analyses of the associations between cardioprotective diet scores and risk of inflammatory bowel disease stratified by Townsend deprivation index, education, and physical activity; Supplementary Table S6. Subgroup analyses of the associations between cardioprotective diet scores and risk of inflammatory bowel disease stratified by smoking status and alcohol-drinking status; Supplementary Table S7. Sensitivity analyses of the associations between cardioprotective diet scores and risk of inflammatory bowel disease when further adjusted for other food groups, the Charlson Comorbidity Index, or exclusion of participants with colorectal cancer at baseline; Supplementary Table S8. Sensitivity analyses of the associations between cardioprotective diet scores and risk of inflammatory bowel disease when further adjusted for medication use, serum C-reactive protein, or reprocessing of missing covariates with multiple imputations.

## References

[1] Jairath V., Feagan B.G. (2020). Global burden of inflammatory bowel disease. Lancet Gastroenterol. Hepatol..

[2] Kaplan G.G., Windsor J.W. (2021). The four epidemiological stages in the global evolution of inflammatory bowel disease. Nat. Rev. Gastroenterol. Hepatol..

[3] Khalili H., Chan S.S.M., Lochhead P., Ananthakrishnan A.N., Hart A.R., Chan A.T. (2018). The role of diet in the aetiopathogenesis of inflammatory bowel disease. Nat. Rev. Gastroenterol. Hepatol..

[4] Khalili H., Håkansson N., Chan S.S., Chen Y., Lochhead P., Ludvigsson J.F., Chan A.T., Hart A.R., Olén O., Wolk A. (2020). Adherence to a Mediterranean diet is associated with a lower risk of later-onset Crohn’s disease: Results from two large prospective cohort studies. Gut.

[5] Lewis J.D., Abreu M.T. (2017). Diet as a Trigger or Therapy for Inflammatory Bowel Diseases. Gastroenterology.

[6] Lichtenstein A.H., Appel L.J., Vadiveloo M., Hu F.B., Kris-Etherton P.M., Rebholz C.M., Sacks F.M., Thorndike A.N., Van Horn L., Wylie-Rosett J. (2021). 2021 Dietary Guidance to Improve Cardiovascular Health: A Scientific Statement From the American Heart Association. Circulation.

[7] Han H., Cao Y., Feng C., Zheng Y., Dhana K., Zhu S., Shang C., Yuan C., Zong G. (2022). Association of a Healthy Lifestyle With All-Cause and Cause-Specific Mortality Among Individuals With Type 2 Diabetes: A Prospective Study in UK Biobank. Diabetes Care.

[8] Lourida I., Hannon E., Littlejohns T.J., Langa K.M., Hyppönen E., Kuzma E., Llewellyn D.J. (2019). Association of Lifestyle and Genetic Risk With Incidence of Dementia. JAMA.

[9] Theiss A.L., Fruchtman S., Lund P.K. (2004). Growth factors in inflammatory bowel disease: The actions and interactions of growth hormone and insulin-like growth factor-I. Inflamm. Bowel Dis..

[10] Łykowska-Szuber L., Rychter A.M., Dudek M., Ratajczak A.E., Szymczak-Tomczak A., Zawada A., Eder P., Lesiak M., Dobrowolska A., Krela-Kaźmierczak I. (2021). What Links an Increased Cardiovascular Risk and Inflammatory Bowel Disease? A Narrative Review. Nutrients.

[11] Gilly A., Park Y.C., Png G., Barysenka A., Fischer I., Bjørnland T., Southam L., Suveges D., Neumeyer S., Rayner N.W. (2020). Whole-genome sequencing analysis of the cardiometabolic proteome. Nat. Commun..

[12] Ananthakrishnan A.N., Khalili H., Konijeti G.G., Higuchi L.M., de Silva P., Korzenik J.R., Fuchs C.S., Willett W.C., Richter J.M., Chan A.T. (2013). A prospective study of long-term intake of dietary fiber and risk of Crohn’s disease and ulcerative colitis. Gastroenterology.

[13] Mozaffari H., Daneshzad E., Larijani B., Bellissimo N., Azadbakht L. (2020). Dietary intake of fish, n-3 polyunsaturated fatty acids, and risk of inflammatory bowel disease: A systematic review and meta-analysis of observational studies. Eur. J. Nutr..

[14] Piovani D., Danese S., Peyrin-Biroulet L., Nikolopoulos G.K., Lytras T., Bonovas S. (2019). Environmental Risk Factors for Inflammatory Bowel Diseases: An Umbrella Review of Meta-analyses. Gastroenterology.

[15] Dong C., Chan S.S.M., Jantchou P., Racine A., Oldenburg B., Weiderpass E., Heath A.K., Tong T.Y.N., Tjønneland A., Kyrø C. (2022). Meat Intake Is Associated with a Higher Risk of Ulcerative Colitis in a Large European Prospective Cohort Studyø. J. Crohn’s Colitis.

[16] Bradbury K.E., Young H.J., Guo W., Key T.J. (2018). Dietary assessment in UK Biobank: An evaluation of the performance of the touchscreen dietary questionnaire. J. Nutr. Sci..

[17] Mozaffarian D. (2016). Dietary and Policy Priorities for Cardiovascular Disease, Diabetes, and Obesity: A Comprehensive Review. Circulation.

[18] Said M.A., Verweij N., van der Harst P. (2018). Associations of Combined Genetic and Lifestyle Risks With Incident Cardiovascular Disease and Diabetes in the UK Biobank Study. JAMA Cardiol..

[19] Xia B., Yang M., Nguyen L.H., He Q., Zhen J., Yu Y., Di M., Qin X., Lu K., Kuo Z.C. (2021). Regular Use of Proton Pump Inhibitor and the Risk of Inflammatory Bowel Disease: Pooled Analysis of 3 Prospective Cohorts. Gastroenterology.

[20] Fu T., Chen H., Chen X., Sun Y., Xie Y., Deng M., Hesketh T., Wang X., Chen J. (2022). Sugar-sweetened beverages, artificially sweetened beverages and natural juices and risk of inflammatory bowel disease: A cohort study of 121,490 participants. Aliment Pharmacol. Ther..

[21] Patterson E. (2005). Guidelines for Data Processing and Analysis of the International Physical Activity Questionnaire (IPAQ)-Short and Long Forms. https://biobank.ndph.ox.ac.uk/showcase/ukb/docs/ipaq_analysis.pdf..

[22] Quan H., Sundararajan V., Halfon P., Fong A., Burnand B., Luthi J.C., Saunders L.D., Beck C.A., Feasby T.E., Ghali W.A. (2005). Coding algorithms for defining comorbidities in ICD-9-CM and ICD-10 administrative data. Med. Care.

[23] O’Quigley J., Flandre P. (1994). Predictive capability of proportional hazards regression. Proc. Natl. Acad. Sci. USA.

[24] Jannasch F., Nickel D.V., Bergmann M.M., Schulze M.B. (2022). A New Evidence-Based Diet Score to Capture Associations of Food Consumption and Chronic Disease Risk. Nutrients.

[25] Abrahami D., Douros A., Yin H., Yu O.H.Y., Renoux C., Bitton A., Azoulay L. (2018). Dipeptidyl peptidase-4 inhibitors and incidence of inflammatory bowel disease among patients with type 2 diabetes: Population based cohort study. BMJ.

[26] Pearson T.A., Mensah G.A., Alexander R.W., Anderson J.L., Cannon R.O., Criqui M., Fadl Y.Y., Fortmann S.P., Hong Y., Myers G.L. (2003). Markers of inflammation and cardiovascular disease: Application to clinical and public health practice: A statement for healthcare professionals from the Centers for Disease Control and Prevention and the American Heart Association. Circulation.

[27] Heeschen C., Dimmeler S., Hamm C.W., Fichtlscherer S., Boersma E., Simoons M.L., Zeiher A.M. (2003). Serum level of the antiinflammatory cytokine interleukin-10 is an important prognostic determinant in patients with acute coronary syndromes. Circulation.

[28] Lo C.H., Lochhead P., Khalili H., Song M., Tabung F.K., Burke K.E., Richter J.M., Giovannucci E.L., Chan A.T., Ananthakrishnan A.N. (2020). Dietary Inflammatory Potential and Risk of Crohn’s Disease and Ulcerative Colitis. Gastroenterology.

[29] Peters V., Bolte L., Schuttert E.M., Andreu-Sánchez S., Dijkstra G., Weersma R.K., Campmans-Kuijpers M.J.E. (2022). Western and Carnivorous Dietary Patterns are Associated with Greater Likelihood of IBD Development in a Large Prospective Population-based Cohort. J. Crohn’s Colitis.

[30] Vasseur P., Dugelay E., Benamouzig R., Savoye G., Lan A., Srour B., Hercberg S., Touvier M., Hugot J.P., Julia C. (2021). Dietary Patterns, Ultra-processed Food, and the Risk of Inflammatory Bowel Diseases in the NutriNet-Santé Cohort. Inflamm. Bowel Dis..

[31] Vargas Robles H., Citalán Madrid A.F., García Ponce A., Silva Olivares A., Shibayama M., Betanzos A., Del Valle Mondragón L., Nava P., Schnoor M. (2016). Experimental Colitis Is Attenuated by Cardioprotective Diet Supplementation That Reduces Oxidative Stress, Inflammation, and Mucosal Damage. Oxid. Med. Cell Longev..

[32] Moro T., Tinsley G., Pacelli F.Q., Marcolin G., Bianco A., Paoli A. (2021). Twelve Months of Time-restricted Eating and Resistance Training Improves Inflammatory Markers and Cardiometabolic Risk Factors. Med. Sci. Sports Exerc..

[33] Sutton E.F., Beyl R., Early K.S., Cefalu W.T., Ravussin E., Peterson C.M. (2018). Early Time-Restricted Feeding Improves Insulin Sensitivity, Blood Pressure, and Oxidative Stress Even without Weight Loss in Men with Prediabetes. Cell Metab..

[34] Wu T., Wang G., Xiong Z., Xia Y., Song X., Zhang H., Wu Y., Ai L. (2022). Probiotics Interact With Lipids Metabolism and Affect Gut Health. Front. Nutr..

[35] Singh S., Dulai P.S., Zarrinpar A., Ramamoorthy S., Sandborn W.J. (2017). Obesity in IBD: Epidemiology, pathogenesis, disease course and treatment outcomes. Nat. Rev. Gastroenterol. Hepatol..

[36] Deleu S., Machiels K., Raes J., Verbeke K., Vermeire S. (2021). Short chain fatty acids and its producing organisms: An overlooked therapy for IBD?. EBioMed..

[37] Masters R.C., Liese A.D., Haffner S.M., Wagenknecht L.E., Hanley A.J. (2010). Whole and refined grain intakes are related to inflammatory protein concentrations in human plasma. J. Nutr..

[38] Roager H.M., Vogt J.K., Kristensen M., Hansen L.B.S., Ibrügger S., Mærkedahl R.B., Bahl M.I., Lind M.V., Nielsen R.L., Frøkiær H. (2019). Whole grain-rich diet reduces body weight and systemic low-grade inflammation without inducing major changes of the gut microbiome: A randomised cross-over trial. Gut.

[39] Parolini C. (2019). Effects of Fish n-3 PUFAs on Intestinal Microbiota and Immune System. Mar. Drugs.

[40] Wilson A., Teft W.A., Morse B.L., Choi Y.H., Woolsey S., DeGorter M.K., Hegele R.A., Tirona R.G., Kim R.B. (2015). Trimethylamine-N-oxide: A Novel Biomarker for the Identification of Inflammatory Bowel Disease. Dig. Dis. Sci..

[41] Freeman K., Ryan R., Parsons N., Taylor-Phillips S., Willis B.H., Clarke A. (2021). The incidence and prevalence of inflammatory bowel disease in UK primary care: A retrospective cohort study of the IQVIA Medical Research Database. BMC Gastroenterol..

[42] Pasvol T.J., Horsfall L., Bloom S., Segal A.W., Sabin C., Field N., Rait G. (2020). Incidence and prevalence of inflammatory bowel disease in UK primary care: A population-based cohort study. BMJ Open.

[43] Jones G.R., Lyons M., Plevris N., Jenkinson P.W., Bisset C., Burgess C., Din S., Fulforth J., Henderson P., Ho G.T. (2019). IBD prevalence in Lothian, Scotland, derived by capture-recapture methodology. Gut.

[44] Cantoro L., Di Sabatino A., Papi C., Margagnoni G., Ardizzone S., Giuffrida P., Giannarelli D., Massari A., Monterubbianesi R., Lenti M.V. (2017). The Time Course of Diagnostic Delay in Inflammatory Bowel Disease Over the Last Sixty Years: An Italian Multicentre Study. J. Crohn’s Colitis.

