# Supplementary material for: Greater Adherence to Cardioprotective Diet Can Reduce Inflammatory Bowel Disease Risk: A Longitudinal Cohort Study
Source: Nutrients. 2022 Sep 29;14(19):4058. doi: 10.3390/nu14194058 (PMC9573093; doi:10.3390/nu14194058)
Supplement: Supplementary file 1 [file nutrients-14-04058-s001.zip › nutrients-1943518-supplementary.pdf]

Greater Adherence to Cardioprotective Diet can Reduce Inflammatory Bowel Disease Risk: A Longitudinal Cohort Study

Tian Fu, Shuyu Ye, Yuhao Sun, Lintao Dan, Xiaoyan Wang, Jie Chen

Catalog

Supplementary Table S1. Definition and classification of covariates 2

Supplementary Table S2. Association between cardioprotective diet and risk of Crohn's disease and ulcerative colitis 3

Supplementary Table S3. Association between each component of cardioprotective diet and risk of IBD in models with single food group and all seven food groups mutually adjusted 4

Supplementary Table S4. Subgroup analyses for the associations between cardioprotective diet scores and risk of inflammatory bowel disease stratified by sex, age, and ethnicity 5

Supplementary Table S5. Subgroup analyses for the associations between cardioprotective diet scores and risk of inflammatory bowel disease stratified by Townsend deprivation Index, education, and physical activity 6

Supplementary Table S6. Subgroup analyses for the associations between cardioprotective diet scores and risk of inflammatory bowel disease stratified by smoking status and alcohol drinking status 7

Supplementary Table S7. Sensitivity analyses for the associations between cardioprotective diet scores and risk of inflammatory bowel disease when further adjusted for other food groups, Charlson Comorbidity Index or excluded participants with colorectal cancer at baseline 8

Supplementary Table S8. Sensitivity analyses for the associations between cardioprotective diet scores and risk of inflammatory bowel disease when further adjusted for medication use, CRP or reprocessed missing covariates with multiple imputations 9

Supplementary Table S1. Definition and classification of covariates

Covariates	Description
**Age**	It can be obtained by the date of birth and the date of the first assessment in the recruitment
**Sex**	Female and male
**Ethnicity**	We classified the variable handlings into **White races** and **other races**.
**Education**	We classified the variable handlings into **College and above** (College or University degree) and **High school and below** (A levels/Advanced Subsidiary levels or equivalent, Ordinary levels/General Certification of Secondary Education or equivalent, Certification of Secondary Education or equivalent, National Vocational Qualification or Higher National Diploma or Higher National Certificate or equivalent, other professional qualifications, and none of the above)
**Townsend deprivation index**	Townsend deprivation index calculated immediately before participant joining UK Biobank. Based on the preceding national census output areas and composed of unemployment, non-car ownership, non-home ownership, and household crowding. Each participant is assigned a score corresponding to the output area in which their postcode is located. We divided all participants into thirds equally: **High deprivation**, **moderate deprivation**, and **low deprivation**.
**Smoking status**	We classified the variable handlings into **previous smokers**, **current smokers**, and **non-smokers**.
**Alcohol status**	We classified the variable handlings into **Non-current drinkers and current drinkers**.
**Physical activity**	Based on International Physical Activity Questionnaire (IPAQ), we classified the variable handlings into **high** physical activity, **moderate** physical activity, and **low** physical activity.
**Body mass index**	Body mass index can be obtained by the weight (kg) and height (m) as recorded by the UK Biobank。
**Other food group**	Including vegetable oils, sugar-sweetened beverages, and dairy products.
**C-reactive protein (**mg/L**)**	Measured by immunoturbidimetric - high sensitivity analysis on a Beckman Coulter AU5800
**Charlson Comorbidity Index**	Charlson Comorbidity Index[1] was calculated by 17 comorbidities including myocardial infarction, congestive heart failure, peripheral vascular disease, cerebrovascular disease, dementia, chronic pulmonary disease, rheumatic disease, peptic ulcer disease, mild liver disease, diabetes without chronic complication, diabetes with chronic complication, hemiplegia or paraplegia, renal disease, any malignancy, including lymphoma and leukemia, except malignant neoplasm of skin, moderate or severe liver disease, metastatic solid tumor, acquired immunodeficiency syndrome according to International Classification of Disease (ICD) codes, measuring comorbid disease status.
**Medication use**	Including nonsteroidal anti-inflammatory drugs (NSAIDs), proton pump inhibitors (PPI) and antibiotics collected from the touchscreen questionnaire and verbal interview at baseline.

1. Quan H, Sundararajan V, Halfon P, et.al. Coding algorithms for defining comorbidities in ICD-9-CM and ICD-10 administrative data. Med Care. 2005 Nov;43(11):1130-9. doi: 10.1097/01.mlr.0000182534.19832.83. PMID: 16224307.

Supplementary Table S2. Association between cardioprotective diet and risk of Crohn's disease and ulcerative colitis

	Cases	Person-year	Crohn's disease		Cases	Person-year	Ulcerative colitis	
			HR (95% CI)[1]	*P*-value			HR (95% CI)	*P*-value
**Score of 0-1**	72	389,518	Ref		163	390,203	Ref	
**Score of 2**	138	786,105	0.96 (0.72, 1.28)	0.792	286	787,162	0.91 (0.75, 1.10)	0.342
**Score of 3**	205	1,328,602	0.86 (0.66, 1.13)	0.293	427	1,330,209	0.85 (0.71, 1.02)	0.076
**Score of 4**	221	1,601,931	0.78 (0.60, 1.03)	0.08	509	1,603,788	0.87 (0.73, 1.04)	0.134
**Score of 5-7**	215	1,734,697	**0.72 (0.55, 0.95)**	**0.022**	481	1,736,575	**0.79 (0.65, 0.95)**	**0.011**
***P*-trend**				**0.002**				**0.014**

Abbreviations: CI, confidence interval; HR, hazard ratio.

[1] Cox model adjusted for sex, age, age-squared, ethnicity, TDI, education, smoking, alcohol drinking, physical activity, and BMI

Supplementary Table S3. Association between each component of cardioprotective diet and risk of IBD in models with single food group and all seven food groups mutually adjusted

Component	Intake goal	Models adjusted for single food groups [1]		Models mutually adjusted for seven food groups [2]	
		HR (95% CI)	*P*-value	HR (95% CI)	*P*-value
**Fruits**	<3 servings/day	Ref		Ref	
	≥3 servings/day	0.88 (0.81, 0.95)	**0.001**	0.90 (0.83, 0.98)	**0.010**
**Vegetables**	<3 servings/day	Ref		Ref	
	≥3 servings/day	0.86 (0.78, 0.94)	**0.001**	0.88 (0.80, 0.97)	**0.013**
**Whole grains**	<3 servings/day	Ref		Ref	
	≥3 servings/day	0.87 (0.77, 0.98)	**0.019**	0.89 (0.79, 1.01)	0.064
**Fish**	<2 servings/day	Ref		Ref	
	≥2 servings/day	0.95 (0.88, 1.03)	0.218	0.99 (0.91, 1.07)	0.744
**Refined grains**	>2 servings/day	Ref		Ref	
	≤2 servings/day	0.91 (0.83, 1.01)	0.069	0.96 (0.87, 1.06)	0.376
**Processed meat**	>1 serving/day	Ref		Ref	
	≤1 serving/day	0.99 (0.91, 1.07)	0.766	1.00 (0.92, 1.09)	0.943
**Unprocessed meat**	>2 servings/day	Ref		Ref	
	≤2 servings/day	1.04 (0.94, 1.16)	0.401	1.04 (0.94, 1.16)	0.413

Abbreviations: CI, confidence interval; HR, hazard ratio.

[1] HR was calculated by the cox model adjusted for single food group, age, age squared, sex, ethnicity, TDI, education, smoking, alcohol drinking, physical activity, and BMI. For example, if the exposure is fruits, HR will be calculated by the cox model adjusted for fruits, age, age squared, sex, ethnicity, TDI, education, smoking, alcohol drinking, physical activity, and BMI.

[2] HR was calculated by the cox model when mutually adjusted for seven food groups (fruits, vegetables, whole grains, fish, refined grains, processed meats, unprocessed meats), age, age squared, sex, ethnicity, TDI, education, smoking, alcohol drinking, physical activity, and BMI.

Supplementary Table S4. Subgroup analyses for the associations between cardioprotective diet scores and risk of inflammatory bowel disease stratified by sex, age, and ethnicity

	**Stratified by sex** [1]		**Stratified by age** [2]		**Stratified by ethnicity** [3]	
	**Female HR (95% CI)**	**Male HR (95% CI)**	**Age ≤ 60 HR (95% CI)**	**Age > 60 HR (95% CI)**	**White HR (95% CI)**	**Other HR (95% CI)**
**P-interaction**		0.90		0.37		0.13
**Score of 0-1**	Ref	Ref	Ref	Ref	Ref	Ref
**Score of 2**	0.92 (0.68, 1.24)	0.92 (0.76, 1.11)	0.92 (0.75, 1.13)	0.94 (0.72, 1.21)	0.92 (0.78, 1.08)	1.25 (0.50, 3.10)
**Score of 3**	0.86 (0.65, 1.13)	0.83 (0.69, 1.00)	0.88 (0.72, 1.06)	0.82 (0.64, 1.05)	**0.84 (0.72, 0.98)**	1.22 (0.51, 2.91)
**Score of 4**	0.85 (0.65, 1.12)	**0.82 (0.68, 0.99)**	0.93 (0.77, 1.12)	**0.74 (0.58, 0.95)**	**0.85 (0.72, 0.99)**	0.95 (0.39, 2.27)
**Score of 5-7**	0.76 (0.58, 1.01)	**0.77 (0.63, 0.94)**	**0.80 (0.66, 0.98)**	**0.73 (0.57, 0.93)**	**0.74 (0.63, 0.87)**	1.44 (0.61, 3.41)
***P*-trend**	**0.013**	**0.004**	0.053	**0.001**	**<0.001**	0.456

Abbreviations: CI, confidence interval; HR, hazard ratio.

[1] Cox model adjusted for age, age-squared, ethnicity, TDI, education, smoking, alcohol drinking, physical activity, and BMI

[2] Cox model adjusted for sex, ethnicity, TDI, education, smoking, alcohol drinking, physical activity, and BMI

[3] Cox model adjusted for age, age-squared, sex, TDI, education, smoking, alcohol drinking, physical activity, and BMI

Supplementary Table S5. Subgroup analyses for the associations between cardioprotective diet scores and risk of inflammatory bowel disease stratified by Townsend deprivation Index, education, and physical activity

	**Stratified by TDI [1]**			**Stratified by education [2]**		**Stratified by physical activity [3]**		
	**High HR (95% CI)**	**Moderate HR (95% CI)**	**Low HR (95% CI)**	**College and above HR (95% CI)**	**High School and below HR (95% CI)**	**High HR (95% CI)**	**Moderate HR (95% CI)**	**Low HR (95% CI)**
**P-interaction**			0.54		0.91			0.60
**Score of 0-1**	Ref	Ref	Ref	Ref	Ref	Ref	Ref	Ref
**Score of 2**	0.84 (0.66, 1.07)	1.04 (0.78, 1.39)	0.95 (0.69, 1.30)	0.92 (0.62, 1.37)	0.93 (0.78, 1.11)	1.09 (0.81, 1.48)	0.79 (0.59, 1.07)	1.01 (0.71, 1.46)
**Score of 3**	0.84 (0.67, 1.06)	0.82 (0.62, 1.09)	0.91 (0.67, 1.23)	0.96 (0.67, 1.38)	**0.83 (0.70, 0.98)**	0.85 (0.63, 1.15)	0.81 (0.62, 1.07)	0.88 (0.62, 1.25)
**Score of 4**	0.82 (0.65, 1.03)	0.95 (0.72, 1.25)	0.78 (0.58, 1.06)	0.89 (0.62, 1.28)	**0.83 (0.70, 0.98)**	0.89 (0.67, 1.19)	0.79 (0.60, 1.03)	0.89 (0.63, 1.27)
**Score of 5-7**	**0.74 (0.59, 0.94)**	0.79 (0.60, 1.05)	0.79 (0.58, 1.07)	0.82 (0.57, 1.18)	**0.75 (0.63, 0.90)**	0.77 (0.58, 1.03)	0.79 (0.60, 1.05)	0.79 (0.54, 1.15)
***P*-trend**	**0.024**	0.051	**0.029**	0.145	**0.001**	**0.007**	0.275	0.121

Abbreviations: CI, confidence interval; HR, hazard ratio.
[1] Cox model adjusted for age, age-squared, sex, ethnicity, education, smoking, alcohol drinking, physical activity, and BMI
[2] Cox model adjusted for age, age-squared, sex, ethnicity, TDI, smoking, alcohol drinking, physical activity, and BMI
[3] Cox model adjusted for age, age-squared, sex, ethnicity, TDI, education, smoking, alcohol drinking, and BMI

Supplementary Table S6. Subgroup analyses for the associations between cardioprotective diet scores and risk of inflammatory bowel disease stratified by smoking status and alcohol drinking status

	Stratified by smoking status [1]			Stratified by alcohol drinking status [2]	
	**Never HR (95% CI)**	**Previous HR (95% CI)**	**Current HR (95% CI)**	**Non-current HR (95% CI)**	**Current HR (95% CI)**
**P-interaction**			0.10		0.54
**Score of 0-1**	Ref	Ref	Ref	Ref	Ref
**Score of 2**	0.82 (0.62, 1.08)	0.99 (0.77, 1.27)	0.98 (0.71, 1.35)	0.86 (0.50, 1.49)	0.94 (0.79, 1.11)
**Score of 3**	0.90 (0.70, 1.16)	0.84 (0.66, 1.07)	0.78 (0.56, 1.07)	0.93 (0.56, 1.55)	**0.85 (0.72, 0.99)**
**Score of 4**	0.91 (0.71, 1.17)	0.82 (0.64, 1.03)	0.73 (0.52, 1.02)	0.92 (0.56, 1.53)	**0.84 (0.71, 0.98)**
**Score of 5-7**	0.89 (0.69, 1.15)	**0.66 (0.52, 0.85)**	0.72 (0.50, 1.04)	0.92 (0.56, 1.52)	**0.75 (0.64, 0.88)**
***P*-trend**	0.968	**<0.001**	**0.014**	0.997	**<0.001**

Abbreviations: CI, confidence interval; HR, hazard ratio.

[1] Cox model adjusted for age, age-squared, sex, ethnicity, TDI, education, alcohol drinking, physical activity, and BMI

[2] Cox model adjusted for age, age-squared, sex, ethnicity, TDI, education, smoking, physical activity, and BMI

Supplementary Table S7. Sensitivity analyses for the associations between cardioprotective diet scores and risk of inflammatory bowel disease when further adjusted for other food groups, Charlson Comorbidity Index or excluded participants with colorectal cancer at baseline

	**Further adjusted for other food groups [1]**		**Further adjusted for baseline Charlson Comorbidity Index**		**Excluded participants with colorectal cancer at baseline (N = 480,273)**	
	**HR (95%CI)**	***P*-value**	**HR (95%CI)**	***P*-value**	**HR (95%CI)**	***P*-value**
**Score of 0-1**	Ref		Ref		Ref	
**Score of 2**	0.92 (0.78, 1.08)	0.312	0.93 (0.79, 1.09)	0.373	0.93 (0.79, 1.09)	0.354
**Score of 3**	0.84 (0.73, 0.98)	**0.030**	0.86 (0.73, 1.00)	**0.044**	0.86 (0.74, 1.00)	**0.046**
**Score of 4**	0.83 (0.72, 0.97)	**0.019**	0.85 (0.73, 0.98)	**0.030**	0.84 (0.72, 0.98)	**0.027**
**Score of 5-7**	0.76 (0.65, 0.89)	**0.001**	0.77 (0.66, 0.90)	**0.001**	0.77 (0.66, 0.90)	**0.001**
***P*-trend**		**<0.001**		**<0.001**		**<0.001**

Abbreviations: CI, confidence interval; HR, hazard ratio.

[1] Other food groups include sugar-sweetened beverages, vegetable oils, and dairy.

Supplementary Table S8. Sensitivity analyses for the associations between cardioprotective diet scores and risk of inflammatory bowel disease when further adjusted for medication use, CRP or reprocessed missing covariates with multiple imputations

	**Further adjusted for medication use [1]**		**Further adjusted for CRP**		**Reprocessed missing covariates using multiple imputations**	
	**HR (95%CI)**	***P*-value**	**HR (95%CI)**	***P*-value**	**HR (95%CI)**	***P*-value**
**Score of 0-1**	Ref		Ref		Ref	
**Score of 2**	0.93 (0.79, 1.09)	0.372	0.93 (0.79, 1.09)	0.376	0.98 (0.81, 1.18)	0.835
**Score of 3**	0.86 (0.74, 1.00)	**0.047**	0.86 (0.74, 1.00)	0.052	0.85 (0.71, 1.02)	0.075
**Score of 4**	0.85 (0.73, 0.99)	**0.035**	0.85 (0.73, 0.99)	**0.040**	0.87 (0.73, 1.03)	0.107
**Score of 5-7**	0.78 (0.67, 0.91)	**0.001**	0.78 (0.67, 0.91)	**0.002**	0.80 (0.67, 0.96)	**0.015**
***P*-trend**		**<0.001**		**0.001**		**0.003**

Abbreviations: CI, confidence interval; HR, hazard ratio; CRP, C-reactive protein.

[1]Medication use including nonsteroidal anti-inflammatory drugs, proton pump inhibitors and antibiotics.
